# GRIN2B alleviates mid‐gestational sevoflurane exposure‐induced early differentiation of rat neural stem cells by interacting with KIF17

**DOI:** 10.1002/ccs3.70024

**Published:** 2025-06-24

**Authors:** Mengyuan Li, Yan Hu, Zhonggui Cheng, Qianqian Li

**Affiliations:** ^1^ Department of Anesthesiology and Operation Medical Center of Anesthesiology and Pain The First Affiliated Hospital of Nanchang University Nanchang China

**Keywords:** differentiation, GRIN2B, KIF17, mid‐trimester, neural stem cells, sevoflurane

## Abstract

General anesthetic exposure during pregnancy has neurotoxic effects on the developing brain, causing long‐term cognitive dysfunction in the offspring. Sevoflurane exposure during mid‐gestation results in premature differentiation of neural stem cells (NSCs), being the crucial factor affecting normal hippocampal functions and contributing to neurocognitive impairment. However, the related molecular mechanism remains unclear. For in vivo assays, pregnant rats were exposed to 3% sevoflurane once on gestational day 14 (G14) or 3 times on G13, 14, and 15 (2 h per day). For in vitro assays, primary rat NSCs were isolated from fetal hippocampus tissues at 24 and 72 h after birth and on postnatal day 28. NSCs were transfected with GRIN2B or KIF17 overexpression plasmids before exposure to 4.1% sevoflurane for one or three consecutive days (2 h per day). Multiple sevoflurane exposures during the mid‐trimester triggered NSC premature differentiation and decreased GRIN2B and KIF17 expression in the hippocampus of offspring rats and primary rat NSCs. GRIN2B or KIF17 overexpression attenuated sevoflurane‐induced NSC premature differentiation. GRIN2B interacted with KIF17, and KIF17 silencing reversed the inhibition of GRIN2B overexpression on NSC early differentiation. GRIN2B alleviates NSC premature differentiation induced by repeated mid‐gestational sevoflurane exposure via interaction with KIF17.

## Graphical abstract

GRIN2B suppresses early differentiation of neural stem cells induced by repeated mid‐gestational sevoflurane exposure and promotes their proliferation by interacting with KIF17.

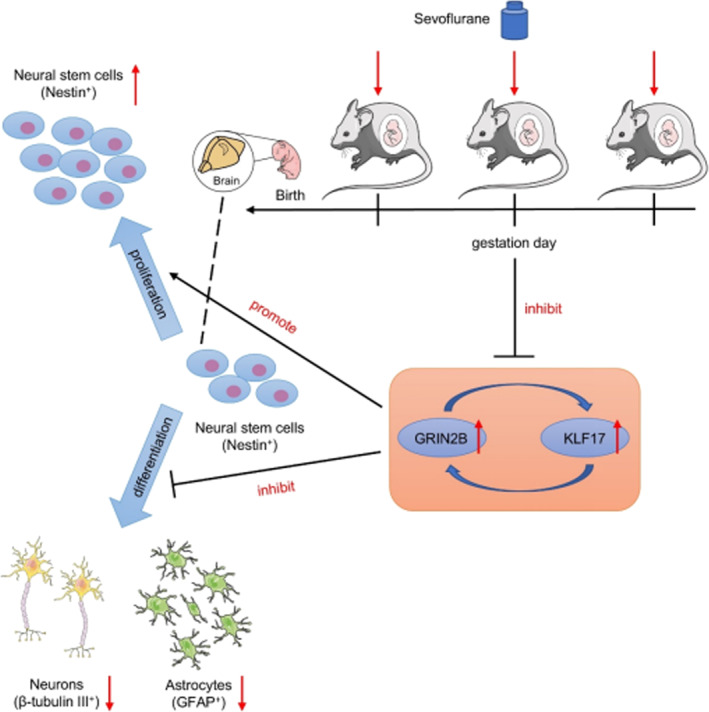

## 1 INTRODUCTION

In recent years, fetal surgery has played an increasingly important role in the second trimester with the advancements in intrauterine fetal therapy, which increases the number of pregnant women undergoing general anesthesia during this period.^1^ The developing brain is influenced by a variety of factors, including genetics, environment, and nutrition, from conception to maturity.^2^ A large body of research evidence suggests that general anesthetic exposure has neurotoxic effects on the developing brain, leading to long‐term cognitive dysfunction.^3^ Animal experimental data show that commonly used anesthetics and sedatives can accelerate neuronal apoptosis and cause neurodegenerative changes in the mammalian brain, and as a result, thereby causing a decline in learning ability.^4^, ^5^ Sevoflurane is the most commonly used anesthetic in clinical practice.^6^ Several studies have demonstrated that exposure of pregnant mammals to inhalational sevoflurane has significant effects on brain development and cognitive function in offspring. For example, prolonged or repeated exposure to inhalational sevoflurane in mid‐pregnancy mice induces neuroinflammation, synaptic loss, and caspase activation, thereby resulting in acute neurotoxicity in the brain tissues of fetal mice as well as learning and memory impairment in offspring mice after birth.^7^ Maternal sevoflurane anesthesia impairs neurological functions in adult offspring by inducing aberrant proliferation and differentiation of neural progenitors in the fetal prefrontal cortex (PFC) through disturbing cell cycle dynamics.^8^ Therefore, it is critical for anesthesiologists not only to provide adequate and effective concentrations of anesthetic drugs but also to consider the short‐ and long‐term effects of fetal exposure to anesthetic drugs.

To date, research on the neurodevelopmental effects of maternal exposure to anesthetics has focused on changes in the hippocampus of the fetus and offspring.^9^ As a part of the brain, the hippocampus plays an important role in learning and memory formation.^10^ The mid‐gestational period is critical for the development of the fetal hippocampus, during which stage neural stem cells (NSCs) undergo high proliferation and differentiation.^11^ NSCs are a class of cells with self‐renewal ability and multidirectional differentiation potential.^12^ During the development of the hippocampus, NSCs first maintain their own number through self‐renewal and then continuously proliferate and differentiate to generate different types of cells, including neurons and glial cells, which provide the cellular basis for maintaining normal hippocampal function.^13^ In addition, NSCs may be involved in the repair of hippocampal damage, helping to restore the function of damaged neurons and neural circuit.^14^ Several studies suggest that the proliferation of hippocampal NSCs during the second trimester is pivotal for hippocampal‐dependent learning.^15^, ^16^ Besides, it was reported that the in vitro self‐renewal ability of cultured NSCs was suppressed after treatment with anesthetic agents.^17^ Despite the proliferation capacity of NSCs, the proliferation process is disrupted if they differentiate early to neurons, which leads to early reduction of neurons and affects normal hippocampal functions. Previously, sevoflurane exposure during the mid‐trimester of pregnancy was confirmed to suppress proliferation, induce early apoptosis, and cause premature differentiation of NSCs, which are considered crucial factors affecting normal hippocampal functions and contributing to neurocognitive impairment.^18^ Accordingly, it is necessary to further explore the mechanism underlying maternal sevoflurane exposure‐induced early differentiation of NSCs.

N‐Methyl‐D‐aspartic acid receptors (NMDARs) are widely expressed in neurons and consist of different subunits, among which GRIN2 subunits A–D vary according to brain region and developmental time window, and GRIN2B (also known as NR2B) is present in embryonic NMDARs.^19^ GRIN2B is widely distributed in the central nervous system, especially in the synaptic regions of the brain, playing an important role in neural development, synaptic plasticity, learning, and memory.^20^ Mutations in the GRIN2B gene can lead to abnormal function of the GRIN2B protein, which in turn affects the signaling of the neurotransmitter glutamate, thereby affecting neural development and synaptic plasticity.^21^ It has been revealed that the abnormal function of the GRIN2B protein is related to the onset and progression of neurodevelopmental disorders.^22^ The majority of individuals with GRIN2B‐associated neurodevelopmental disorders exhibit intellectual disability, developmental delay, schizophrenia, epilepsy, autism spectrum disorder, microcephaly, cortical visual impairment, and malformations of cortical development in the clinic.^23^ However, whether GRIN2B contributes to offspring neurological impairment due to mid‐pregnancy anesthesia remains unknown. KIF17 is a multifunctional, homodimeric microtubule motor that plays a vital role in regulating transcriptional activators, transport of RNA granules, vesicular transport, and the building of sensory cilia.^24^ Recent studies have revealed that KIF17 is expressed in neurons and participates in modulating the dendrite‐targeted transport of NR2B.^25^ KIF17 binds to the NR2B cargo system via the mLin10 scaffolding protein and makes the NMDARs functional at the cell surface.^25^ The previous study has demonstrated that KIF17 can mediate offspring learning and memory deficits induced by maternal anesthesia in mid‐pregnancy and that KIF17 impairs learning and memory in offspring rats by inhibiting the translocation of NR2B to the neuronal cell membrane.^26^ Similarly, the detailed mechanisms are unclear. Importantly, a possible interaction between GRIN2B and KIF17 has been discovered through bioinformatics analysis.

Neurodevelopment in rats is similar to that in humans, with 14 days of gestation corresponding to mid‐gestation in humans. Although the neurodevelopmental time frames of rats and humans are different, NSC proliferation and apoptosis in the developing hippocampus are similar. Therefore, we used rats at 14 days of gestation for our experiments. We respectively evaluated the effects of GRIN2B and KIF17 on early differentiation of rat NSCs induced by mid‐gestational sevoflurane exposure and whether the interaction between GRIN2B and KIF17 is involved in this process.

## 2 MATERIALS AND METHODS

### 2.1 Animals and sevoflurane exposure

Healthy adult Sprague–Dawley (SD) rats (SPF grade, 380–420 g) purchased from Vital River Laboratories (Beijing, China) were raised in an environment under natural lighting with a temperature of 20–25°C, with free access to food and water. Two to three female rats were confined in a cage with one male rat to mate freely. The presence of a positive vaginal smear on the second day was considered indicative of gestation day 0 (G0). Pregnant rats were placed into a 30% oxygen plastic chamber and randomized into the SEV × 1 group (exposure to 3% sevoflurane for 2 h on gestational day 14 [G14]), the SEV × 3 group (exposure to 3% sevoflurane for 2 h on G13, G14, and G15), and the control group (without exposure to sevoflurane), with 7 rats in each group. The fetal hippocampus tissues were obtained at 24 and 72 h after cesarean section on G15 and on postnatal day 28. The experiment was approved by the Animal Ethics Committee of the Hubei Provincial Center for Disease Control and Prevention (No. 202320089).

### 2.2 Primary rat NSC isolation

Primary rat NSCs were isolated from the hippocampus of fetal rat brains as previously described.^27^ In brief, pregnant rats (G15) were anesthetized by inhalation of isoflurane (4% for induction and 1%–3% for maintenance), and the fetal rats were recovered from the uterus. An Olympus dissecting microscope was used to isolate the hippocampi of fetal rats, which were immersed in Hanks' balanced salt solution (HBSS; H1020; Solarbio, Shanghai, China). A 2‐mL Pasteur pipette was used to gently triturate the tissue fragments to acquire single‐cell suspension. NSCs were expanded as neurospheres in Dulbecco's modified Eagle medium (DMEM)/F12 (#PM150312; Procell, Wuhan, China) that contained 2% B27 without vitamin A (#60704ES10; Yeason, Shanghai, China), 1% penicillin‐streptomycin (#ZY90307; Zeye, Shanghai, China), 20 ng/mL epidermal growth factor (#CYT‐669; AmyJet Scientific, Wuhan, China), and 20 ng/mL basic fibroblast growth factor (#abs45152529; Absin, Shanghai, China). NSCs were plated at a density of 1 × 10^5^ cells/mL in 10‐cm Petri dishes and maintained at 37°C in a 5% CO_2_ incubator. The culture medium was half replaced with fresh DMEM/F12 medium every other day, and the cells were digested with Accutase (#40506ES60; Yeason). When the spheres were clearly visible after 5–7 days, the NSCs were passaged using TrypLE Express (#12604013; Gibco, USA). All the following experiments were performed using NSCs from passages 2–4. To induce NSC differentiation, rat NSCs were plated (6 × 10^5^ cells/well) onto coverslips precoated with 0.25% poly‐D‐lysine (#C0312; Beyotime, Shanghai, China) using differentiation medium of DMEM/F12 supplemented with a half concentration of growth factors.

### 2.3 EdU assay

A BeyoClick™EdU‐594 kit (#C0078L; Beyotime) was used to determine the proliferation capability of NSCs. In short, NSCs were cultured for 12 h in differentiation medium supplemented with 10‐μM EdU. After fixation in 4% paraformaldehyde, cells were incubated with 3% bovine serum albumin (BSA; #YB01050; Yubo, Shanghai, China) and 0.3% Triton X‐100 (#T8200; Solarbio) in phosphate‐buffered saline (PBS; #AWR0213a; Abiowell, Changsha, China), followed by staining with a click additive solution provided in the kit and nuclear staining with DAPI (#HXSJ‐021134; Jisskang, Qingdao, China). Finally, the images were captured under a fluorescent microscope (Leica, Germany), and the number of EdU‐positive cells was counted using ImageJ software.

### 2.4 Sevoflurane exposure and cell transfection for in vitro assays

For in vitro experiments, rat NSCs were isolated from hippocampus tissues obtained at 24 and 72 h after cesarean section on G15 and on postnatal day 28. NSCs were inoculated into 24‐well plates that were placed into an incubator containing 95% air and 5% CO_2_, and sevoflurane concentration was detected using a gas monitor (Drӓger, Germany). Cells were randomized into control, Sevo × 1 (exposure to 4.1% sevoflurane for one day, 2 h per day), Sevo × 3 (exposure to 4.1% sevoflurane for three consecutive days, 2 h per day), Sevo × 3 + vector (exposure to 4.1% sevoflurane for three consecutive days and transfection with empty control vector pcDNA3.1), Sevo × 3 + GRIN2B (exposure to 4.1% sevoflurane for three consecutive days and transfection with GRIN2B overexpression pcDNA3.1 vector), Sevo × 3 + KIF17 (exposure to 4.1% sevoflurane for three consecutive days and transfection with GRIN2B overexpression pcDNA3.1 vector), Sevo × 3 + vector + siNC (exposure to 4.1% sevoflurane for three consecutive days and co‐transfection with empty control vector pcDNA3.1 and negative control siRNA vector), and Sevo × 3 + GRIN2B + siKIF17 (exposure to 4.1% sevoflurane for three consecutive days and co‐transfection with GRIN2B overexpression pcDNA3.1 vector and KIF17 silencing siRNA vector) groups. All control groups were kept under the same conditions (95% air and 5% CO_2_) for the same amount of time without exposure to sevoflurane. For cell transfection, NSCs were transfected with the above plasmids for 48 h before exposure to 4.1% sevoflurane by using Lipofectamine 3000 (#L3000‐008, Invitrogen, Carlsbad, CA, USA).

### 2.5 RT‐qPCR

Total RNA from NSCs was extracted with TRIzol reagent (#mlsw‐2443; Mlbio, Shanghai, China) and later converted to cDNA by reverse transcription with HiScript II Q room temperature (RT) SuperMix (#R222‐01; Vazyme, Nanjing, China). The semi‐quantitative reverse transcriptase PCR analysis was conducted with 2‐μg cDNA as the template using SYBR Green qPCR Master Mix (#G3323‐15; Servicebio, Wuhan, China) on the Bio‐Rad CFX Connect Real‐Time System (Bio‐Rad, Hercules, CA, USA). The amplified PCR products were analyzed using 1% agarose gel electrophoresis. Relative expression was quantified by the comparative CT method, with GAPDH as the normalization control. The following primer sequences were used: GRIN2B forward primer (5′‐GGATCTACCAGTCTAACATG‐3′), GRIN2B reverse primer (5′‐GATAGTTAGTGATCCCACTG‐3′); GAPDH forward primer (5′‐AACCTGCCAAGTATGATG‐3′), GAPDH reverse primer (5′‐GGAGTTGCTGTTGAAGTC‐3′).

### 2.6 Western blotting

Rat hippocampus tissues and NSCs were lysed using RIPA lysis buffer (#C1053‐500; Applygen, Beijing, China) added with protease (#ZY80808; Zeye) and phosphatase (#wb5012; Warbio, Nanjing, China) inhibitor cocktails. A BCA protein assay kit (#LM2015; LMAI Bio, Shanghai, China) was used for the protein quantification. Subsequently, a total of 40 μg protein samples were loaded on SDS‐PAGE and transferred onto PVDF membranes, which were immersed in 5% nonfat milk for 2 h at RT and incubated with primary antibodies against nestin (#A11861; 1:1000; ABclonal, Wuhan, China), β‐tubulin III (#A17913; 1:1000; ABclonal), GFAP (#A19058; 1:1000; ABclonal), GRIN2B (#A3056; 1:1000; ABclonal), KIF17 (#A16562; 1:1000; ABclonal), and GAPDH (#AC027; 1:2000; ABclonal) overnight at 4°C. After three washes with TBST, HRP goat anti‐rabbit IgG secondary antibody (#AS014; 1:2000; ABclonal) was added for another 1 h incubation at 37°C. The immunoblot signals were visualized using Odyssey Infrared Imaging Scanner (Odyssey LI‐COR Biosciences, Lincoln, NE, USA), and the band intensities were analyzed using ImageJ software. GAPDH was used as the loading control.

### 2.7 Immunofluorescence staining

Using 4% paraformaldehyde, NSCs and their differentiated cells grown on culture slides were fixed for 1 h at RT. After three washes (5 min/time) with PBS, cells were incubated with 0.3% Triton X‐100 for 10 min for permeabilization and with 5% BSA for 1 h to block of nonspecific binding. Thereafter, cells were incubated overnight at 4°C with primary antibodies against nestin (#A11861; 1:100; ABclonal), β‐tubulin III/Tuj1 (#A17913; 1:200; ABclonal), or GFAP (#A19058; 1:200; ABclonal), followed by 1 h incubation at RT with Alexa Fluor 488‐conjugated (#AS053; 1:100; ABclonal) or Alexa Fluor 594‐labeled (#AS039; 1:100; ABclonal) goat anti‐rabbit IgG secondary antibodies. For the colocalization assay, NSCs were coincubated with the anti‐GRIN2B (#A3056; 1:100; ABclonal) and anti‐KIF17 (#A16562; 1:100; ABclonal) primary antibodies and subsequently stained with Alexa Fluor 488‐labeled and Alexa Fluor 594‐conjugated secondary antibodies. DAPI (1 mg/mL) was used for nuclear staining for 10 min at 4°C protected from light. For tissue staining, rat hippocampus tissues were fixed in 4% formaldehyde, dehydrated using an automatic dehydration device, cleared for 30 min in xylene, embedded in paraffin with 3 cycles (1 h per cycle), and then cut into 5‐μm‐thick sections. After dewaxing with xylene and rehydration with graded alcohol, the sections were permeabilized with 0.1% Triton X‐100 for 3 min and incubated with 5% BSA for 1 h at RT. Then, the sections were incubated with nestin (#A11861; 1:100; ABclonal), β‐tubulin III (#A17913; 1:200; ABclonal), or GFAP (#A19058; 1:200; ABclonal) primary antibody overnight at 4°C and with Alexa Fluor 488‐conjugated secondary antibody (#AS053; 1:100; ABclonal) for 1 h at RT. The fluorescent signals were observed under an LSM710 laser scanning confocal microscope (Carl Zeiss). The number of positive‐stained cells was calculated using ImageJ software.

### 2.8 Immunohistochemical staining

To examine GRIN2B and KIF17 expression in rat hippocampus tissues, the paraffin‐embedded tissues after dewaxing and rehydration were immersed in 3% hydrogen peroxide for 10 min to deactivate internal peroxidase, boiled in sodium citrate solution to repair antigens, and washed three times (5 min each time) using PBS. Thereafter, sections were sealed for 30 min by applying 3% BSA, followed by incubation with anti‐GRIN2B (#FNab05839; FineTest, Wuhan, China) or KIF17 (#A16562; 1:100; ABclonal) primary antibody overnight at 4°C and with secondary antibody linked with HRP (#AS014; 1:100; ABclonal) for 1 h at RT. At last, the sections were placed in 3,3′‐diaminobenzidine (DAB; #S19134; Yuanye, Shanghai, China) dye solution for color development and counterstained with hematoxylin (#S19007; Yuanye). Finally, the mounted sections were photographed under an Olympus BX53 digital microscope, followed by the quantification of positive‐stained cells using ImageJ software.

### 2.9 Co‐immunoprecipitation (Co‐IP) assay

HEK293T cells (#CL‐0005; Procell, Wuhan, China) transiently co‐transfected with HA‐tagged GRIN2B and Flag‐tagged KIF17 were seeded in 100‐mm dishes and lysed with RIPA lysis buffer supplemented with protease and phosphatase inhibitors. Cell lysates were sonicated for 30 s, and the supernatant was collected and kept on ice for 1 h, followed by 2 h of incubation with 5 μg of specific antibody and control IgG plus 50 μL protein A/G magnetic beads (#CSM7005; Chemstan, Wuhan, China) on a shaker at 4°C. After the magnetic beads were washed thrice with PBS, the immunoprecipitates were boiled with SDS loading buffer and subjected to Western blotting analysis.

### 2.10 Statistical analysis

All data were statistically analyzed using SPSS 22.0 software (IBM Corp., Armonk, NY, USA). All values are expressed as the mean ± SD. Significant differences between and among the experimental groups were determined using Student's *t*‐test or one‐way ANOVA followed by the Bonferroni post hoc test. Values of *p* < 0.05 were regarded as statistically significant.

## 3 RESULTS

### 3.1 Repeated maternal exposure to sevoflurane induces early NSC differentiation and decreases GRIN2B and KIF17 expression in fetal brains

**FIGURE 1:**
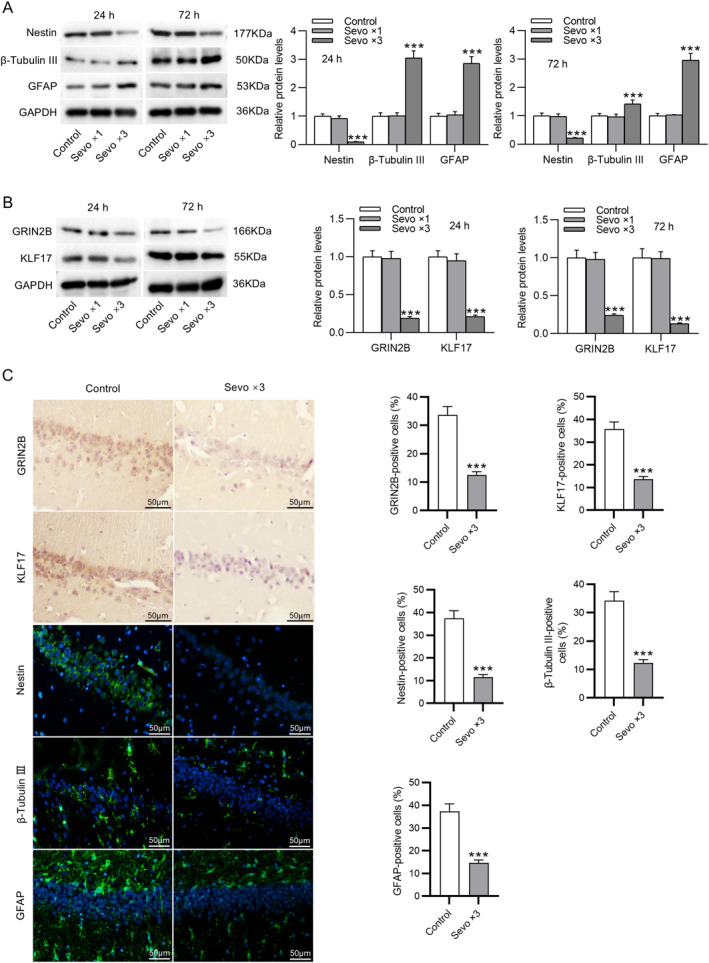
Repeated maternal exposure to sevoflurane induces early neural stem cell differentiation and decreased GRIN2B and KIF17 expression in fetal brains. (A) Measurement of nestin, β‐tubulin III, and GFAP protein levels in the fetal hippocampus at 24 and 72 h after birth following the pregnant rats had received single or repeated exposure to sevoflurane by Western blotting. (B) Examination of GRIN2B and KIF17 protein levels in the fetal hippocampus at 24 and 72 h after birth following single or multiple maternal exposure to sevoflurane through Western blotting. (C) Representative immunohistochemical and immunofluorescence staining images showing GRIN2B, KIF17, nestin, β‐tubulin III, and GFAP expression in the hippocampus tissues of fetal rats at 24 h after birth following repeated maternal exposure to sevoflurane and quantification of positive‐stained cells. Scale bar: 50 μm. *n* = 7. ***p < 0.001.

First, to assess the effects of sevoflurane on early NSC differentiation, the pregnant rats received single or repeated exposure to sevoflurane, and the fetal hippocampus was obtained at 24 and 72 h after birth. The expression of nestin (NSC marker), β‐tubulin III (neuron marker), and GFAP (astrocyte marker) in the fetal hippocampus was examined by Western blotting. We found that repeated maternal exposure to sevoflurane resulted in a marked increment in β‐tubulin III and GFAP levels and a decrement in nestin levels in the fetal hippocampus. Nevertheless, no changes in β‐tubulin III, GFAP, and nestin levels were observed in the hippocampus of fetal rats obtained from pregnant rats that received no or single exposure to sevoflurane (Figure 1A). Besides, GRIN2B and KIF17 protein levels were significantly decreased after multiple maternal exposures to sevoflurane compared to groups not exposed to sevoflurane or exposed only once to sevoflurane (Figure 1B). The results of immunohistochemical and immunofluorescence staining were in line with those of Western blotting, which revealed that GRIN2B, KIF17, and nestin expression was considerably attenuated, whereas β‐tubulin III and GFAP expression was enhanced in the hippocampus of fetal rats at 24 h after birth following repeated maternal exposure to sevoflurane (Figure 1C). Therefore, the reduction in GRIN2B and KIF17 expression might be associated with early NSC differentiation induced by multiple maternal exposures to sevoflurane.

### 3.2 Identification of primary rat NSCs

**FIGURE 2:**
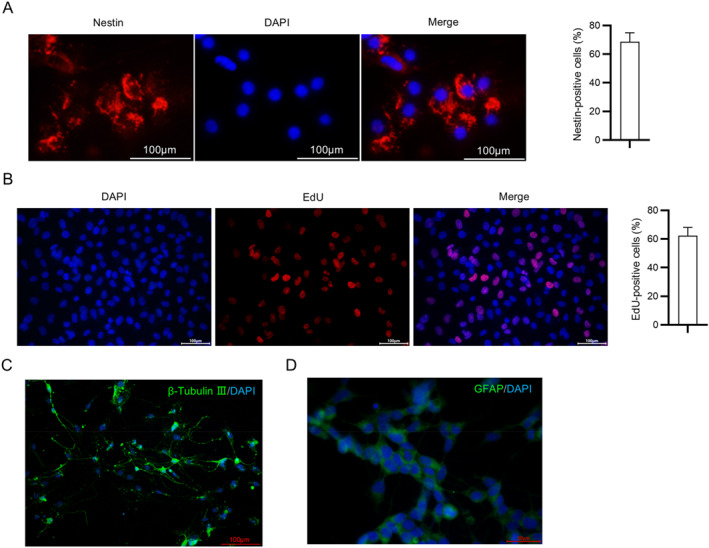
Identification of primary rat NSCs. (A) Representative immunofluorescence staining images for NSC‐specific marker nestin in NSCs. DAPI: nucleus stain. Scale bar: 100 μm. (B) Representative EdU staining images showing the proliferation of NSCs. DAPI: nucleus stain. Scale bar: 100 μm. (C, D) Representative immunofluorescence staining images for neuron marker Tuj1 (scale bar: 100 μm) and astrocyte marker GFAP (scale bar: 50 μm) in NSCs after induction of differentiation. NSCs, neural stem cells.

Next, primary rat NSCs were isolated to determine the potential toxicity of sevoflurane against NSCs. It was observed through immunofluorescence staining that nearly 70% of primary rat NSCs were positive for nestin (Figure 2A). Besides, primary rat NSCs presented potent proliferation capability, as demonstrated by EdU staining (Figure 2B). After induction of differentiation, NSCs were subjected to immunofluorescence staining for neuron and astrocyte markers, which revealed that they could differentiate into Tuj1‐positive neurons and GFAP‐positive astrocytes (Figure 2C–D). Overall, the isolated primary rat NSCs have strong self‐renewal ability and multidirectional potential.

### 3.3 Overexpression of GRIN2B inhibits early NSC differentiation induced by repeated sevoflurane exposure

NSCs were isolated from fetal rat hippocampus 24 or 72 h after birth and then exposed to 4.1% sevoflurane for 2 h per day for one day or three consecutive days. As shown by RT‐qPCR, GRIN2B mRNA expression was remarkably downregulated in NSCs after multiple exposures to sevoflurane (Figure 3A). To determine the influence of GRIN2B overexpression on sevoflurane‐induced early NSC differentiation, NSCs were transfected with GRIN2B overexpression plasmid before repeated exposure to sevoflurane. Western blotting indicated that the reduction in GRIN2B protein levels and elevation in β‐tubulin III and GFAP protein levels in NSCs caused by multiple sevoflurane exposures were reversed after overexpressing GRIN2B. Importantly, no significant difference was observed in GRIN2B, β‐tubulin III, and GFAP protein levels at 24 and 72 h between the control and Sevo × 3 + GRIN2B groups (Figure 3B,C). Consistently, immunofluorescence staining of NSCs isolated from fetal rat hippocampus 24 h after birth demonstrated that overexpressing GRIN2B antagonized sevoflurane‐induced enhancement in β‐tubulin III and GFAP expression (Figure 3D). Although NSCs have proliferation ability, the proliferation process is interrupted if they differentiate early to neurons, which leads to a decrease in the number of neurons in the developing brain over an extended period. EdU staining demonstrated that repeated exposure to sevoflurane prominently decreased EdU incorporation in NSCs, which, however, was overturned by overexpression of GRIN2B (Figure 3E), suggesting that GRIN2B overexpression rescued sevoflurane‐induced decline in NSC proliferation. In addition, NSCs isolated from fetal rat hippocampus 28 days after birth were repeatedly exposed to sevoflurane. We found that GRIN2B and β‐tubulin III protein levels were inhibited, whereas GFAP protein level remained overexpressed after exposure to sevoflurane. Nevertheless, such changes caused by sevoflurane in β‐tubulin III and GFAP protein levels were abolished by overexpression of GRIN2B (Figure 3F). These results indicate that GRIN2B overexpression suppresses early NSC differentiation induced by repeated sevoflurane exposure.

FIGURE 3Overexpression of GRIN2B inhibits early NSC differentiation induced by repeated sevoflurane exposure. (A) Detection of GRIN2B mRNA expression in NSCs (isolated from fetal rat hippocampus 24 or 72 h after birth) following exposure to 4.1% sevoflurane for one or three consecutive days (2 h per day) by RT‐qPCR. **p < 0.01, ***p < 0.001 versus control; ##*p* < 0.01, ###*p* < 0.001 versus Sevo × 3. (B, C) Examination of GRIN2B, β‐tubulin III, and GFAP protein levels in NSCs (isolated from fetal rat hippocampus 24 or 72 h after birth) following transfection with GRIN2B overexpression plasmid and exposure to 4.1% sevoflurane for three consecutive days (2 h per day) through Western blotting. (D) Representative immunofluorescence staining images showing β‐tubulin III and GFAP expression in NSCs (isolated from fetal rat hippocampus 24 h after birth) and quantification of positive‐stained cells. Scale bar: 20 μm. (E) Representative EdU staining images showing the proliferation of NSCs (isolated from fetal rat hippocampus 24 h after birth) and quantification of EdU‐positive cells. Scale bar: 200 μm. (F) Measurement of GRIN2B, β‐tubulin III, and GFAP protein levels in NSCs (isolated from fetal rat hippocampus 28 days after birth) through Western blotting. **p < 0.01, ***p < 0.001 versus control; #*p* < 0.05, ##*p* < 0.01, ###*p* < 0.001 versus Sevo × 3 + vector. NSCs, neural stem cells.

**Figure:**
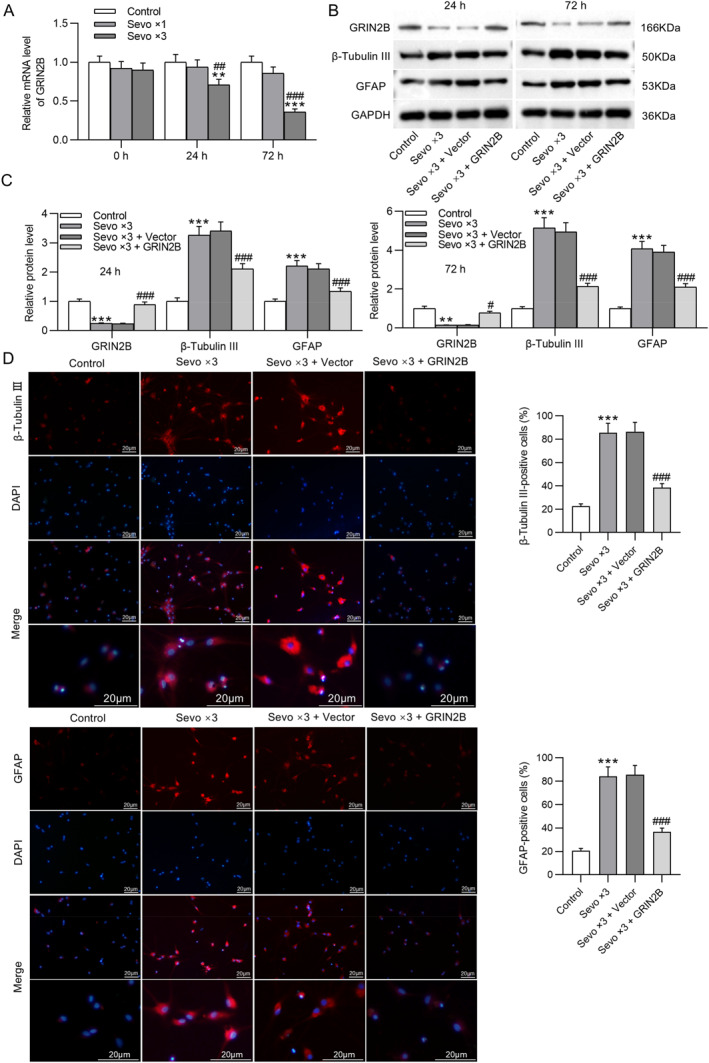

**Figure:**
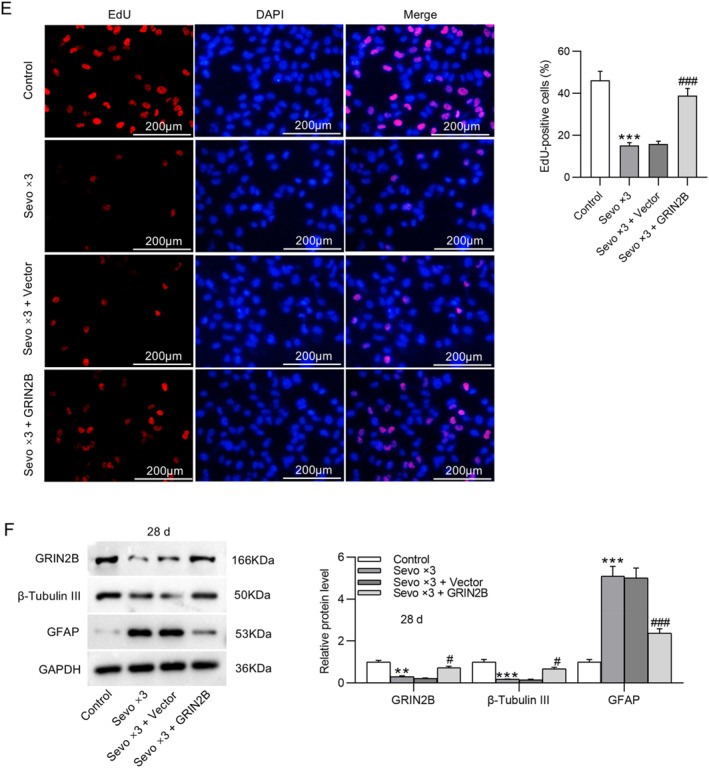

### 3.4 Overexpression of KIF17 suppresses early NSC differentiation caused by multiple sevoflurane exposures

Similarly, whether the upregulation of KIF17 can inhibit early NSC differentiation induced by repeated sevoflurane exposure was also analyzed in NSCs receiving transfection with KIF17 overexpression plasmid and repeated exposure to sevoflurane. As revealed by Western blotting, the sevoflurane‐induced decrease in KIF17 protein levels and increase in β‐tubulin III and GFAP protein levels in NSCs were overturned by overexpression of KIF17. There was no marked significance in KIF17, β‐tubulin III, and GFAP protein levels at 24 and 72 h between the control and Sevo × 3 + KIF17 groups (Figure 4A). The results of immunofluorescence staining of β‐tubulin III and GFAP in NSCs isolated from fetal rat hippocampus 24 h after birth were consistent with those of Western blotting. It was observed that the elevation in β‐tubulin III and GFAP expression caused by repeated exposure to sevoflurane was counteracted by overexpression of KIF17 (Figure 4B). In addition, NSCs isolated from fetal rat hippocampus 28 days after birth were repeatedly exposed to sevoflurane, which resulted in a reduction in KIF17 and β‐tubulin III protein levels and an elevation in GFAP protein levels. However, sevoflurane‐induced changes in KIF17, β‐tubulin III, and GFAP protein levels were offset by KIF17 overexpression (Figure 4C). Meanwhile, KIF17 overexpression reversed the decrease in EdU incorporation in NSCs caused by repeated exposure to sevoflurane (Figure 4D). Accordingly, overexpressing KIF17 hinders early NSC differentiation induced by repeated sevoflurane exposure.

FIGURE 4Overexpression of KIF17 suppresses early NSC differentiation caused by multiple sevoflurane exposures. (A) Assessment of KIF17, β‐tubulin III, and GFAP protein levels in NSCs (isolated from fetal rat hippocampus 24 or 72 h after birth) following transfection with KIF17 overexpression plasmid and exposure to 4.1% sevoflurane for three consecutive days (2 h per day) through Western blotting. (B) Representative immunofluorescence staining images showing β‐tubulin III and GFAP expression in NSCs (isolated from fetal rat hippocampus 24 h after birth) and quantification of positive‐stained cells. Scale bar: 20 μm. (C) Evaluation of KIF17, β‐tubulin III, and GFAP protein levels in NSCs (isolated from fetal rat hippocampus 28 days after birth) by Western blotting. (D) Representative EdU staining images showing the proliferation of NSCs (isolated from fetal rat hippocampus 24 h after birth) and quantification of EdU‐positive cells. Scale bar: 100 μm.*p < 0.05, **p < 0.01, ***p < 0.001 versus control; #*p* < 0.05, ##*p* < 0.01, ###*p* < 0.001 versus Sevo × 3 + vector. NSCs, neural stem cells.

**Figure:**
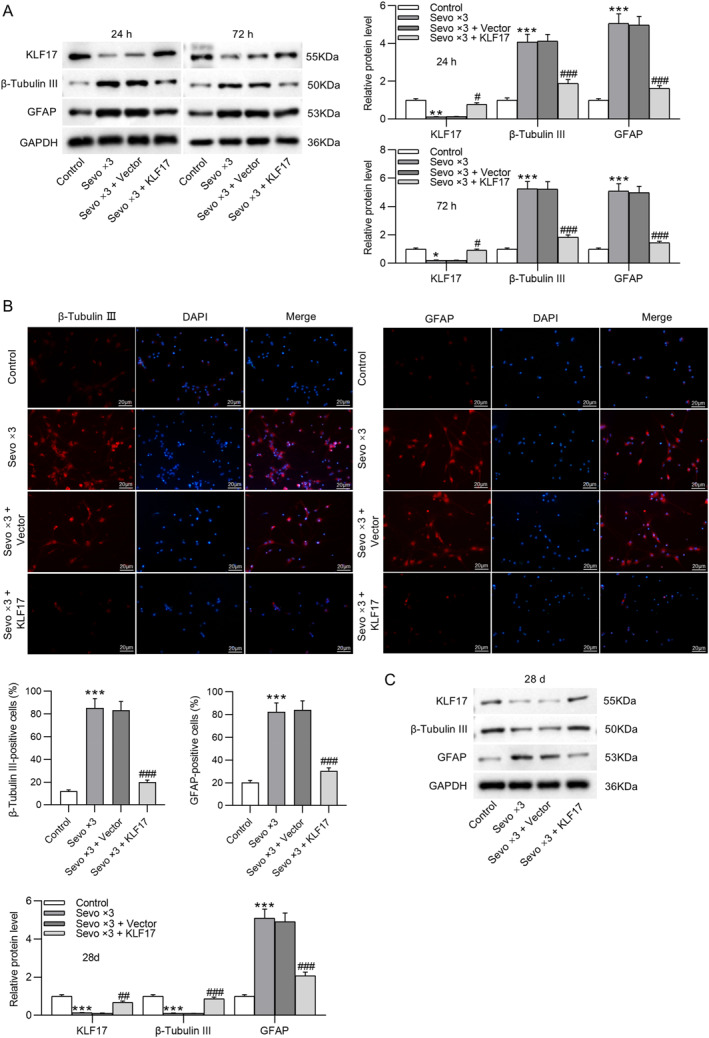

**Figure:**
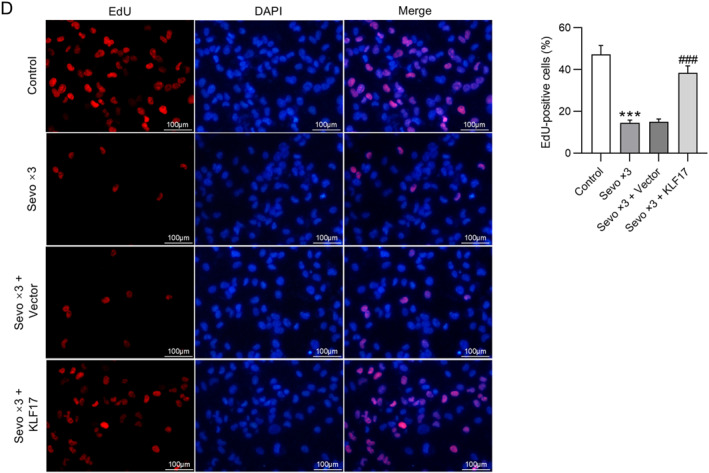

### 3.5 GRIN2B interacts with KIF17 in NSCs

**FIGURE 5:**
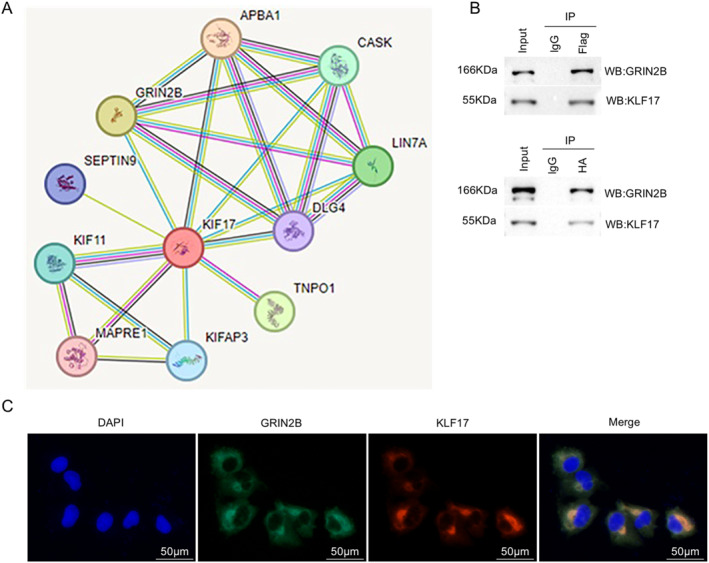
GRIN2B interacts with KIF17 in NSCs. (A) Analysis of the interaction network between GRIN2B and KIF17 using the STRING database. (B) Validation of the interaction between GRIN2B and KIF17 by co‐IP assay using HEK293T cells transfected with HA‐tagged GRIN2B and Flag‐tagged KIF17. (C) Representative immunofluorescence staining images showing GRIN2B and KIF17 localization in NSCs. Scale bar: 50 μm. NSCs, neural stem cells.

According to the STRING database (https://string‐db.org/), KIF17 was predicted to be the potential protein that could interact with GRIN2B (Figure 5A). To further investigate whether the inhibitory effects of GRIN2B on early NSC differentiation are related to the interaction between GRIN2B and KIF17, their binding affinity was validated by co‐IP assay. HA‐tagged GRIN2B and Flag‐tagged KIF17 were transfected into HEK293T cells, and co‐IP analysis proved the strong interaction between GRIN2B and KIF17 (Figure 5B). Moreover, the cytoplasmic colocalization of GRIN2B and KIF17 in NSCs was observed in immunofluorescence staining (Figure 5C). Taken together, the above data indicate that GRIN2B physically associates with KIF17.

### 3.6 KIF17 knockdown reverses the inhibition of GRIN2B on sevoflurane‐induced early NSC differentiation

Finally, rescue experiments were conducted to verify whether GRIN2B represses sevoflurane‐induced early NSC differentiation by interacting with KIF17. NSCs isolated from fetal rat hippocampus 24 or 72 h after birth were co‐transfected with GRIN2B overexpression plasmid and KIF17 silencing siRNA before exposure to sevoflurane for three consecutive days. Western blotting illustrated that the effects of GRIN2B overexpression on sevoflurane‐induced upregulation in β‐tubulin III and GFAP protein levels and downregulation in GRIN2B and KIF17 protein levels in NSCs were further reversed by knockdown of KIF17 (Figure 6A). As expected, KIF17 silencing overturned the suppressive effects of GRIN2B overexpression on sevoflurane‐induced enhancement in β‐tubulin III and GFAP expression in NSCs, as shown by immunofluorescence staining (Figure 6B). Moreover, the promotive effects of GRIN2B overexpression on the proliferation of NSCs after sevoflurane exposure were abolished by knockdown of KIF17 (Figure 6C). These results suggest that GRIN2B curbs sevoflurane‐induced early NSC differentiation by interacting with KIF17.

FIGURE 6KIF17 knockdown reverses the inhibition of GRIN2B on sevoflurane‐induced early NSC differentiation. (A) Measurement of GRIN2B, KIF17, β‐tubulin III, and GFAP protein levels in NSCs (isolated from fetal rat hippocampus 24 or 72 h after birth) following co‐transfection with GRIN2B overexpression plasmid and KIF17 silencing siRNA and exposure to 4.1% sevoflurane for three consecutive days through Western blotting. (B) Representative immunofluorescence staining images showing β‐tubulin III and GFAP expression in NSCs (isolated from fetal rat hippocampus 24 h after birth) and quantification of positive‐stained cells. Scale bar: 20 μm. (C) Representative EdU staining images showing the proliferation of NSCs (isolated from fetal rat hippocampus 24 h after birth) and quantification of EdU‐positive cells. Scale bar: 200 μm. ***p < 0.001 versus control; ###*p* < 0.001 versus Sevo × 3 + vector + siNC; &&&*p* < 0.001 versus Sevo × 3 + GRIN2B. NSCs, neural stem cells.

**Figure:**
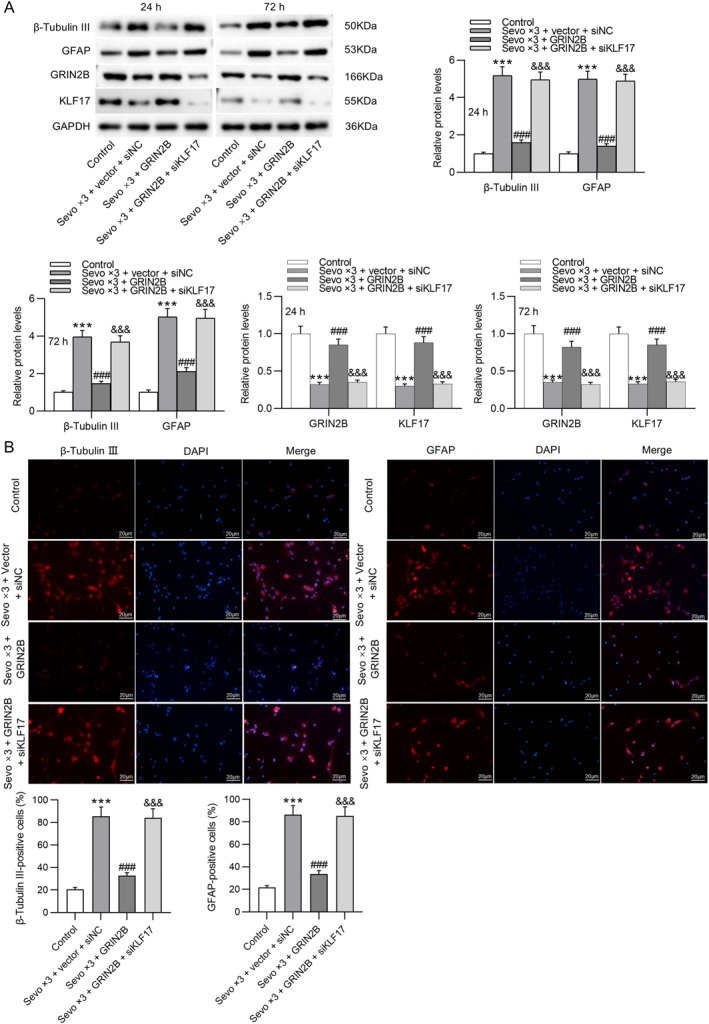

**Figure:**
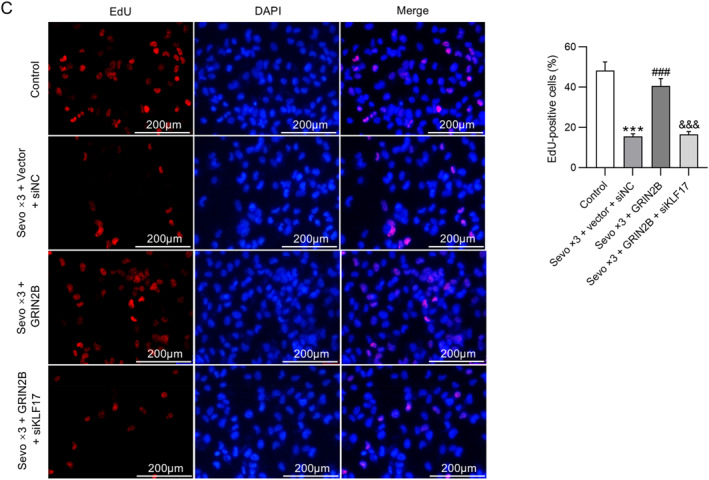

## 4 DISCUSSION

It has been well documented that exposure to anesthesia agents during the mid‐trimester, which is the most common time for nonobstetric surgery, can cause long‐term neurocognitive impairments in developing brains.^28^ In this study, we investigated the novel molecular mechanism underlying the premature differentiation of fetal hippocampal NSCs after mid‐gestational exposure to sevoflurane, which might provide advice for further clinical application. Our results showed that either GRIN2B or KIF17 overexpression markedly suppressed repeated maternal sevoflurane exposure‐induced early differentiation of fetal hippocampal NSCs. Importantly, GRIN2B could interact with KIF17, and silencing of KIF17 reversed the protective effects of GRIN2B against sevoflurane‐induced NSC premature differentiation.

Specific antigen expression is an important indicator in the identification of NSCs, and relevant molecular markers can be verified using immunofluorescence detection. For example, the expression of nestin, which is considered the most representative marker of NSCs, starts to be expressed at the time of neural embryo formation and gradually decreases or even stops completely with the completion of migration and differentiation of NSCs.^29^ NSCs have good differentiation potential and can differentiate spontaneously or be induced to neurons, astrocytes, oligodendrocytes, and so on.^30^ NSCs cultured in serum‐containing medium for about 7 days will spontaneously differentiate into neuronal cells or astrocytes, at which time they can express β‐tubulin III or GFAP markers. β‐Tubulin III is a major component of microtubules in neurons, which is highly expressed during embryonic and postnatal development and plays a key role in proper axon guidance, maturation, and maintenance.^31^ As a neuronal marker, the positive expression of β‐tubulin III is necessary for the identification of neurons.^32^ GFAP is a central nervous system‐specific type III intermediate filament protein involved in cytoskeleton formation and maintenance of its turgor strength.^33^ GFAP is expressed predominantly in astrocytes and is an astrocyte‐specific marker.^34^ In our study, immunofluorescence staining of NSCs with anti‐nestin, anti‐β‐tubulin III, and anti‐GFAP antibodies was performed for the identification of isolated rat NSCs. The results showed that primary rat NSCs expressed nestin and presented potent proliferation capability. After differentiation induction, NSCs expressed both β‐tubulin III (Tuj1) and GFAP, indicating that they can differentiate into β‐tubulin III‐positive neurons and GFAP‐positive astrocytes.

To ensure lifelong maintenance of the hippocampal NSC population and potential, NSCs need to undergo protracted dividing, differentiation, and depletion during early development.^35^ In the nervous system, NSCs must carefully keep a delicate balance between self‐renewal and differentiation, which is essential to ensure the appropriate number of differentiated cell types required for the maintenance of brain homeostasis and development.^36^ It has been demonstrated that cell cycle timing dictates cell fate decisions.^37^ The balance between NSC self‐renewal and differentiation is achieved through controlling cell division.^38^ Failure to properly control cell division may result in abnormal self‐renewal, growth, and differentiation.^38^ Premature neuronal differentiation may lead to an inadequate pool of neural progenitor cells and an imbalance in the distribution of other types of neural cells, hindering neural cell migration, and impairing brain area projections.^39^ Several studies have clarified that premature differentiation of NSCs can negatively affect the developing brain and lead to severe brain developmental disorders. For example, Gogendeau et al. suggested that aneuploidy decreased the number of proliferative NSCs, extended the G1 phase, and caused cell cycle exit and premature differentiation of NSCs, thereby inducing the formation of microcephalic brains.^40^ Yang et al. disclosed that the reduction in the levels of glyoxalase 1 resulted in premature neurogenesis in murine models of maternal diabetes and brought long‐term adverse effects on brain development in offspring.^41^ Hence, deciphering the molecular mechanisms that regulate cell fate and maintain the homeostasis of NSC differentiation is crucial.

Sevoflurane is a halogenated ether inhalation anesthetic drug that is commonly used for the induction and maintenance of general anesthesia.^42^ The analgesic effects of sevoflurane have been proven in many clinical trials.^43^, ^44^ Moreover, preconditioning with sevoflurane at subanesthetic levels was reported to reduce hypoxia‐induced brain damage in a model of intrauterine perinatal asphyxia and exert neuroprotective effects, suggesting that sevoflurane may be effective in preventing neonatal hypoxic‐ischemic encephalopathy.^45^ However, accumulating evidence has revealed that sevoflurane can induce neurotoxicity in the developing brains of rodents and impair the proliferation, self‐renewal, and differentiation of NSCs. For example, Zuo et al. discovered that exposure of postnatal day 7 rats to 2% sevoflurane for 4 h damaged spatial learning and working memory and led to cognitive deficits in rats by reducing the self‐renewal ability of hippocampal NSCs, reduced the number of NSCs, as well as curbed neurogenesis and NSC proliferation.^46^ In the study conducted by Wang et al., pregnant rats at mid‐gestation were exposed to a high concentration (3.5%) of sevoflurane, which led to enhancement in the apoptosis of NSCs and reduction of nestin and SOX2 levels in the fetal brain and postnatal hippocampus and eventually memory and learning dysfunctions in the offspring.^47^ Sevoflurane was also reported to inactivate the Wnt/β‐catenin signaling pathway to induce G0/G1 cell cycle arrest, which triggered premature differentiation of NSCs, inhibited the proliferation of fetal NSCs, and impaired postnatal learning and memory functions.^48^ In addition, Zhang et al. reported that premature differentiation of NSCs could be observed both in the hippocampus of fetal rats after repeated mid‐gestational exposure to 3% sevoflurane and in primary cultured rat NSCs after repeated treatment by 4.1% sevoflurane, which was mediated by the miR‐410‐3p/ATN1 pathway, reducing the reserves, pluripotency, and hypoxia tolerance of hippocampal NSCs.^18^ In our study, we exposed pregnant rats and primary cultured rat NSCs to sevoflurane with the same concentration and duration as described in the study of Zhang et al.^18^ and discovered consistent results. 3% sevoflurane for in vivo assays and 4.1% sevoflurane for in vitro assays have also been used in other relevant studies.^49^, ^50^ Our data revealed that three times maternal exposures to sevoflurane downregulated nestin expression and upregulated β‐tubulin III and GFAP expression in the hippocampus of fetal rats, whereas a single maternal exposure to sevoflurane exerted no significant effects on NSC early differentiation in the fetal hippocampus. Besides, similar changes in nestin, β‐tubulin III, and GFAP expression were observed in the isolated rat hippocampus NSCs after three times exposures to sevoflurane, indicating sevoflurane‐induced premature differentiation of NSCs. To be noted, the early differentiation of NSCs will interrupt their own proliferation process and thereby lead to decreased number of neurons in the developing brain over an extended period. Herein, we found that GFAP remained overexpressed, whereas β‐tubulin III was inhibited following exposure to sevoflurane in NSCs isolated from fetal rat hippocampus 28 days after birth, suggesting the reduction in neuron numbers over an extended period.

NMDARs are a subtype of ionic glutamate receptors, which are unique dual‐gated channel complexes controlled by both membrane potential and other neurotransmitters.^51^ NMDARs are mainly distributed in the presynaptic membrane and posterior membrane of nerve cells in the central and peripheral systems.^52^ NMDARs not only play an important physiological role in the development of the nervous system, such as regulating the survival of neurons, modulating the development of neuronal dendrites and axons, and participating in the formation of synaptic plasticity, but also play a key role in the formation of neuronal circuits.^53^ NMDARs are formed by the assembly of an essential subunit, NR1, and various modulatory NR2 subunits (NR2A‐D).^52^ The NR2B (GRIN2B) subunit is critical for synaptic localization of NMDAR channels and directly participates in enhancing the learning and memory functions.^54^ NMDARs are preferentially inhibited by general anesthetics such as sevoflurane.^55^ In a Xenopus laevis model expressing human GRIN1/GRIN2B receptor channels, inhaled sevoflurane significantly suppressed GRIN1/GRIN2B expression.^56^ The kinesin superfamily proteins (KIFs) are critical for the sorting and delivery of organelles in highly polarized cells including neurons.^57^ Defects in KIFs damage neuronal functions such as neurotransmitter release and action potential propagation.^58^ The previous study has confirmed the function of the molecular motor KIF17 in the active transport and regulation of NR2B in living hippocampal neurons.^59^ KIF17 vesicles specifically enter and move progressively along dendrites, thereby transporting and delivering NR2B subunits within the dendrites.^60^ KIF17 can bind to a protein complex containing mLin10 and the NR2B subunit of NMDARs.^61^ NR2B expression and its synaptic localization can be markedly impaired by KIF17 knockdown or functional blockade.^62^ In contrast, treatment with the NMDAR antagonist can upregulate the expression of NR2B and simultaneously increase the expression of KIF17.^59^ Interestingly, KIF17 was reported to mediate maternal anesthesia‐induced offspring learning and memory deficits in mid‐pregnancy, and KIF17 impaired learning memory in offspring rats through repressing NR2B translocation to the neuronal cell membrane.^26^ In our study, we found that repeated maternal exposure to sevoflurane significantly reduced GRIN2B and KIF17 levels in both hippocampus tissues of fetal rats and fetal hippocampal NSCs. Overexpressing GRIN2B or KIF17 suppressed early NSC differentiation in vitro induced by repeated sevoflurane exposure. Mechanically, the interaction between GRIN2B and KIF17 was predicted by bioinformatics tools and further verified by co‐IP assay. Rescue experiment demonstrated that silencing of KIF17 abolished the inhibitory effects of GRIN2B overexpression on sevoflurane‐triggered NSC premature differentiation.

To be honest, there were some limitations in our study. First, it has been reported that in addition to the duration of exposure, the exposure concentration of anesthetic agents also affects their neurotoxicity. Further studies are required to identify the effects of diverse concentrations of sevoflurane on NSC early differentiation. Second, because the density of cultured NSCs isolated from the hippocampus of fetal rats at 28 days after birth was too low for immunofluorescence staining, we only used Western blotting to detect the expression of β‐tubulin III and GFAP. Third, primary cultured NSCs were used to simulate the in vivo environment, and the mechanisms by which GRIN2B alleviated mid‐gestational sevoflurane exposure‐induced early differentiation of NSCs by interacting with KIF17 were only confirmed in vitro. The in vivo mechanism was not verified due to technical limitations.

**FIGURE 7:**
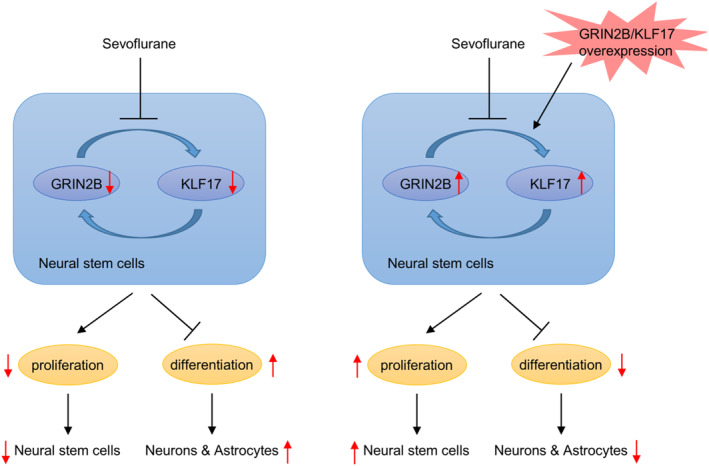
A working model of GRIN2B and KIF17 interaction in regulating proliferation and early differentiation of neural stem cells in response to sevoflurane.

In summary, in vivo and in vitro data from our study demonstrated that multiple exposures to sevoflurane during mid‐trimester pregnancy resulted in premature differentiation of NSCs in the offspring. Importantly, either GRIN2B or KIF17 overexpression effectively alleviated NSC early differentiation caused by maternal sevoflurane exposure. After validating the interaction between GRIN2B and KIF17, we further confirmed that GRIN2B suppressed NSC early differentiation by interacting with KIF17 (Figure 7). The above findings might open novel avenues to further explore the mechanisms of sevoflurane on NSC differentiation.

## AUTHOR CONTRIBUTIONS

Mengyuan Li conceived and designed the experiments. Mengyuan Li, Yan Hu, Zhonggui Cheng, and Qianqian Li carried out the experiments. Mengyuan Li, Yan Hu, Zhonggui Cheng, and Qianqian Li analyzed the data. Mengyuan Li, Yan Hu, Zhonggui Cheng, and Qianqian Li drafted the manuscript. All authors agreed to be accountable for all aspects of the work. All authors have read and approved the final manuscript.

## CONFLICT OF INTEREST STATEMENT

The authors declare no conflicts of interest.

## ETHICS STATEMENT

The experiment was approved by the Animal Ethics Committee of the Hubei Provincial Center for Disease Control and Prevention (No. 202320089).

## CONSENT FOR PUBLICATION

Not Applicable.

## Supporting information

Supporting Information S1

## Data Availability

All data generated or analyzed during this study are available from the corresponding author upon reasonable request.
