# Supplementary material for: GRIN2B alleviates mid‐gestational sevoflurane exposure‐induced early differentiation of rat neural stem cells by interacting with KIF17
Source: J Cell Commun Signal. 2025 Jun 24;19(2):e70024. doi: 10.1002/ccs3.70024 (PMC12187615; doi:10.1002/ccs3.70024)
Supplement: Supplementary file 1 — Supporting Information S1 [file CCS3-19-e70024-s001.pptx]

## Slide 1
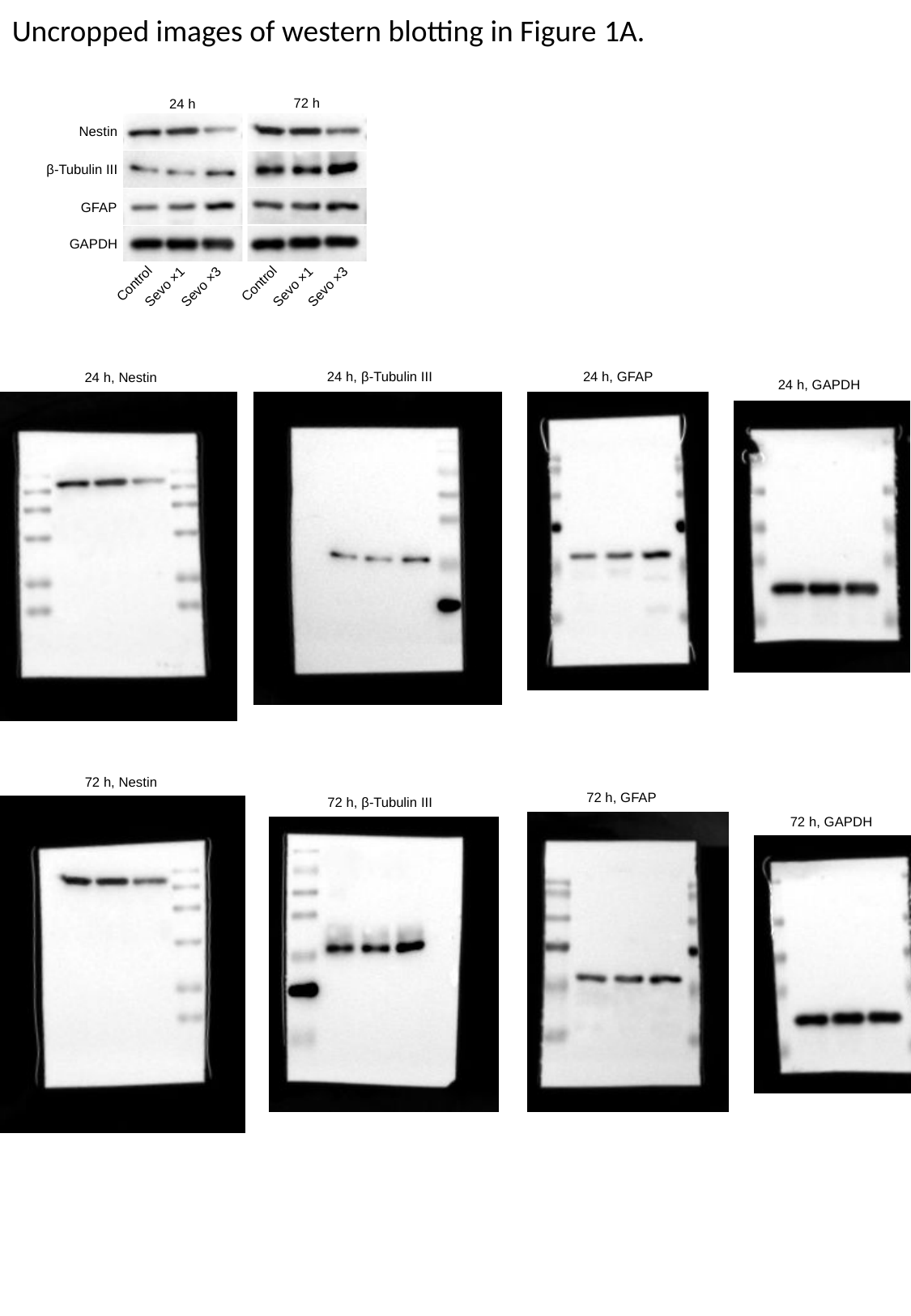

Uncropped images of western blotting in Figure 1A.
72 h
24 h
Nestin
β-Tubulin III
GFAP
GAPDH
Control
Control
Sevo ×1
Sevo ×1
Sevo ×3
Sevo ×3
24 h, β-Tubulin III
24 h, GFAP
24 h, Nestin
24 h, GAPDH
72 h, Nestin
72 h, GFAP
72 h, β-Tubulin III
72 h, GAPDH

## Slide 2
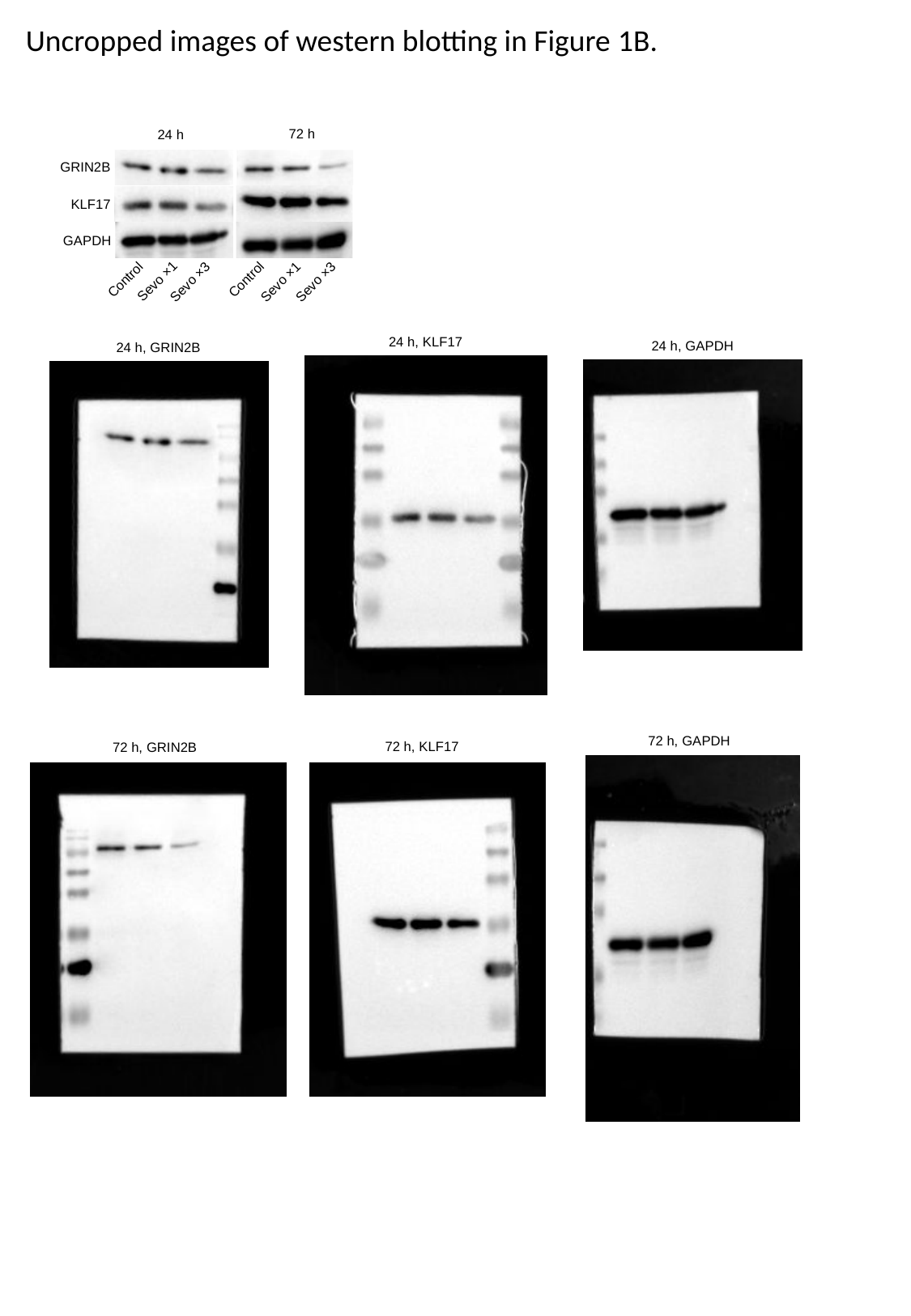

Uncropped images of western blotting in Figure 1B.
72 h
24 h
GRIN2B
KLF17
GAPDH
Control
Control
Sevo ×1
Sevo ×3
Sevo ×3
Sevo ×1
24 h, KLF17
24 h, GAPDH
24 h, GRIN2B
72 h, GAPDH
72 h, KLF17
72 h, GRIN2B

## Slide 3
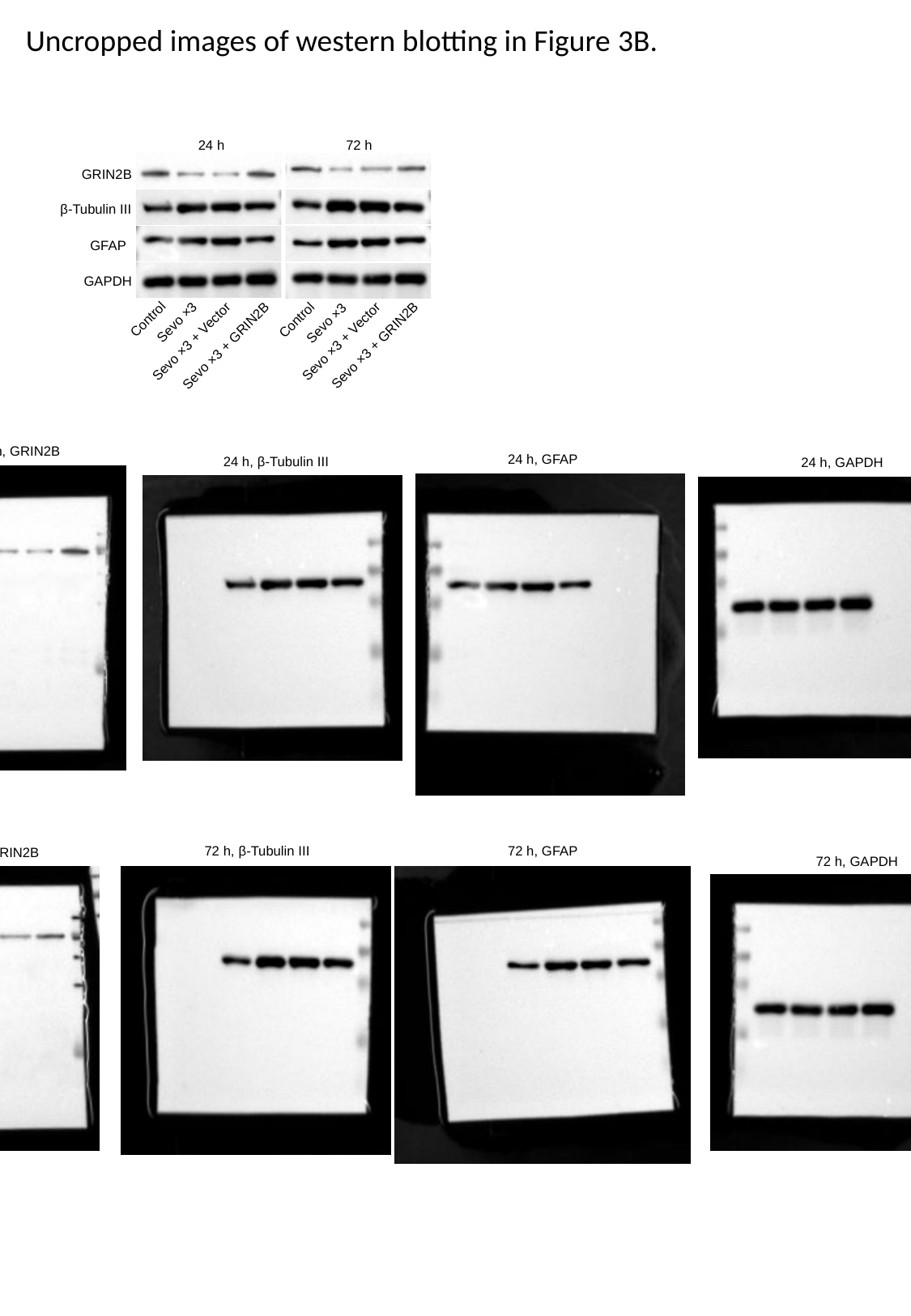

Uncropped images of western blotting in Figure 3B.
24 h
72 h
GRIN2B
β-Tubulin III
GFAP
GAPDH
Control
Control
Sevo ×3
Sevo ×3
Sevo ×3 + Vector
Sevo ×3 + Vector
Sevo ×3 + GRIN2B
Sevo ×3 + GRIN2B
24 h, GRIN2B
24 h, GFAP
24 h, β-Tubulin III
24 h, GAPDH
72 h, GFAP
72 h, β-Tubulin III
72 h, GRIN2B
72 h, GAPDH

## Slide 4
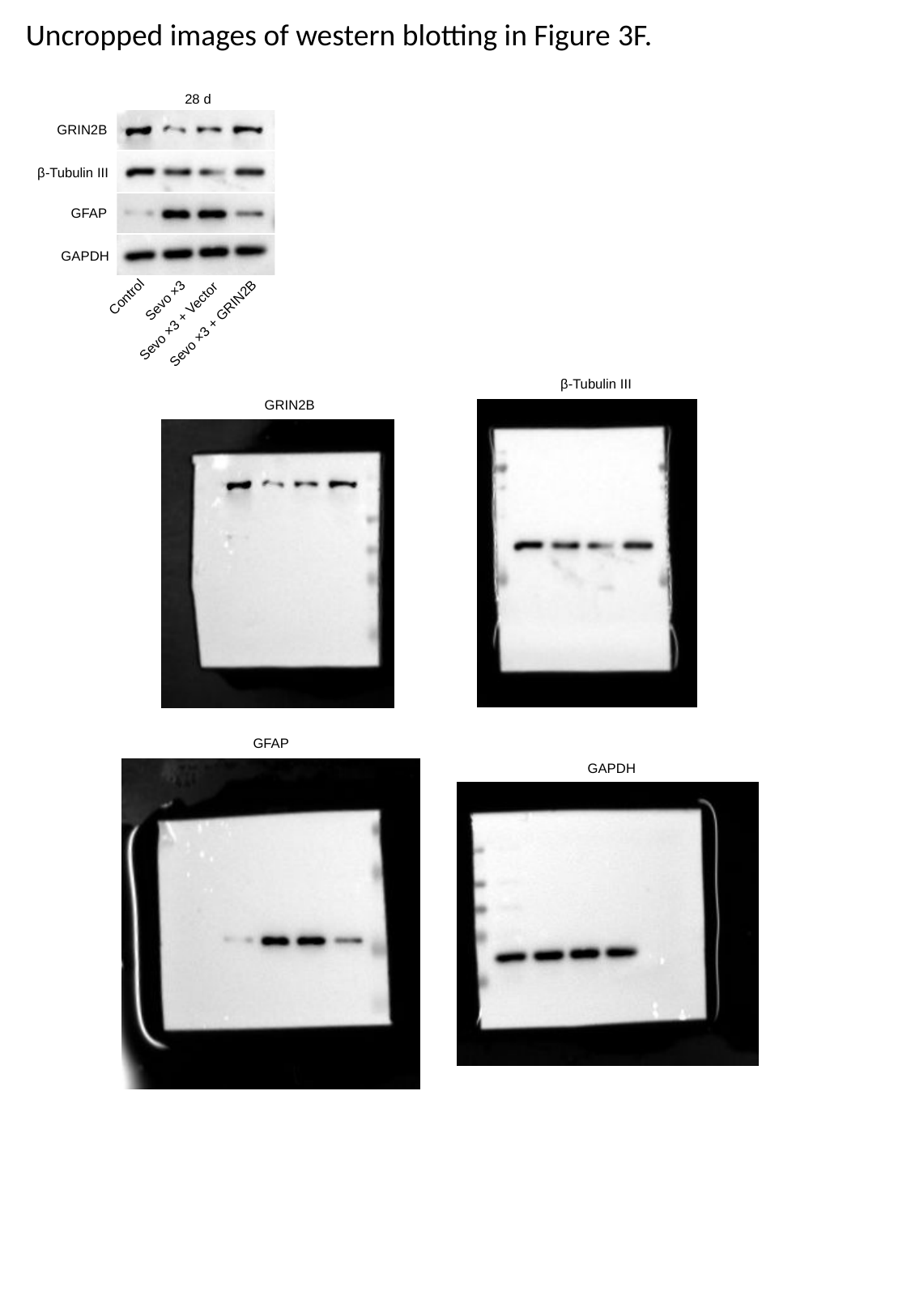

Uncropped images of western blotting in Figure 3F.
28 d
GRIN2B
β-Tubulin III
GFAP
GAPDH
Control
Sevo ×3
Sevo ×3 + Vector
Sevo ×3 + GRIN2B
β-Tubulin III
GRIN2B
GFAP
GAPDH

## Slide 5
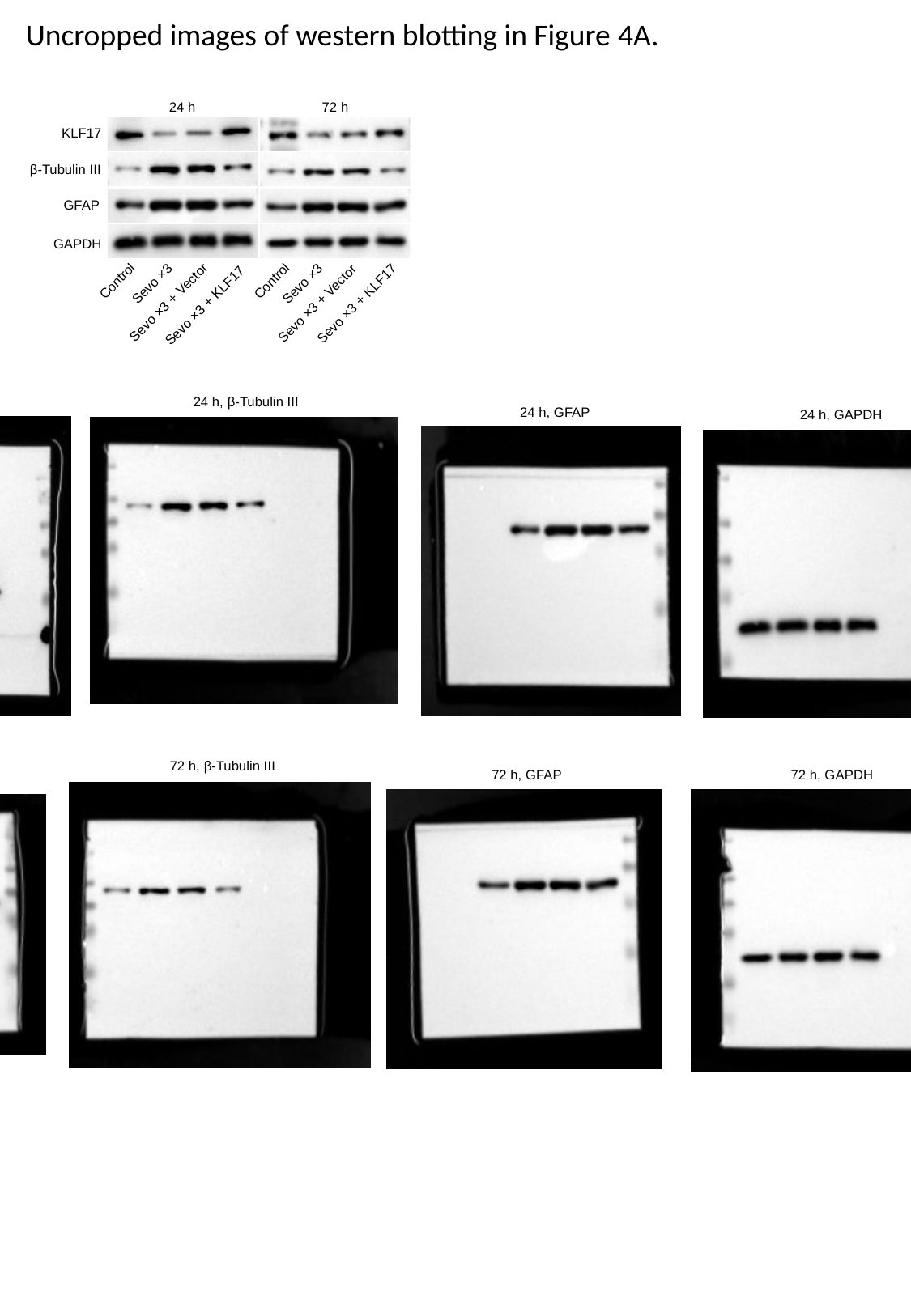

Uncropped images of western blotting in Figure 4A.
24 h
72 h
KLF17
β-Tubulin III
GFAP
GAPDH
Control
Control
Sevo ×3
Sevo ×3
Sevo ×3 + Vector
Sevo ×3 + Vector
Sevo ×3 + KLF17
Sevo ×3 + KLF17
24 h, KLF17
24 h, β-Tubulin III
24 h, GFAP
24 h, GAPDH
72 h, β-Tubulin III
72 h, GAPDH
72 h, GFAP
72 h, KLF17

## Slide 6
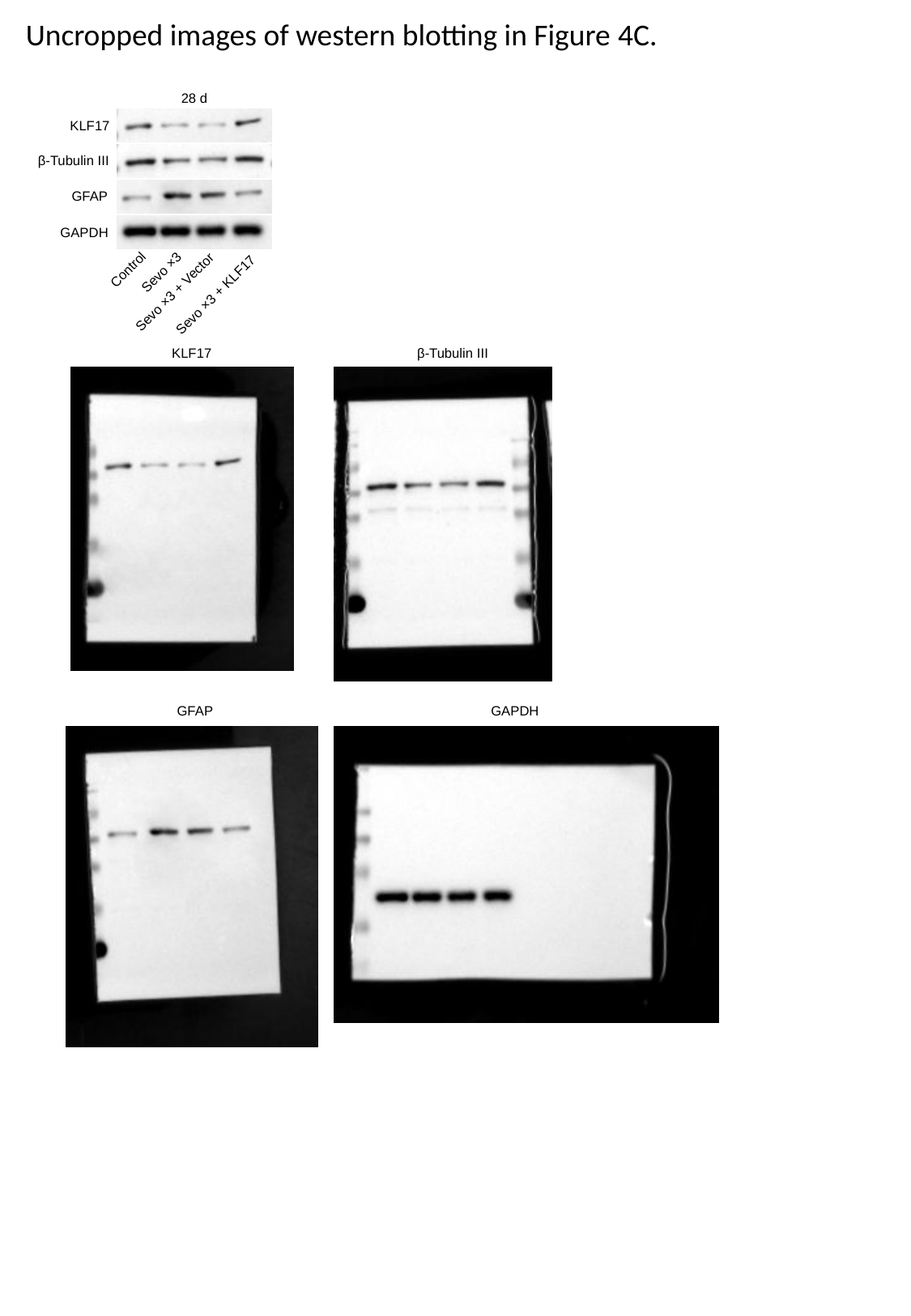

Uncropped images of western blotting in Figure 4C.
28 d
KLF17
β-Tubulin III
GFAP
GAPDH
Control
Sevo ×3
Sevo ×3 + Vector
Sevo ×3 + KLF17
KLF17
β-Tubulin III
GAPDH
GFAP

## Slide 7
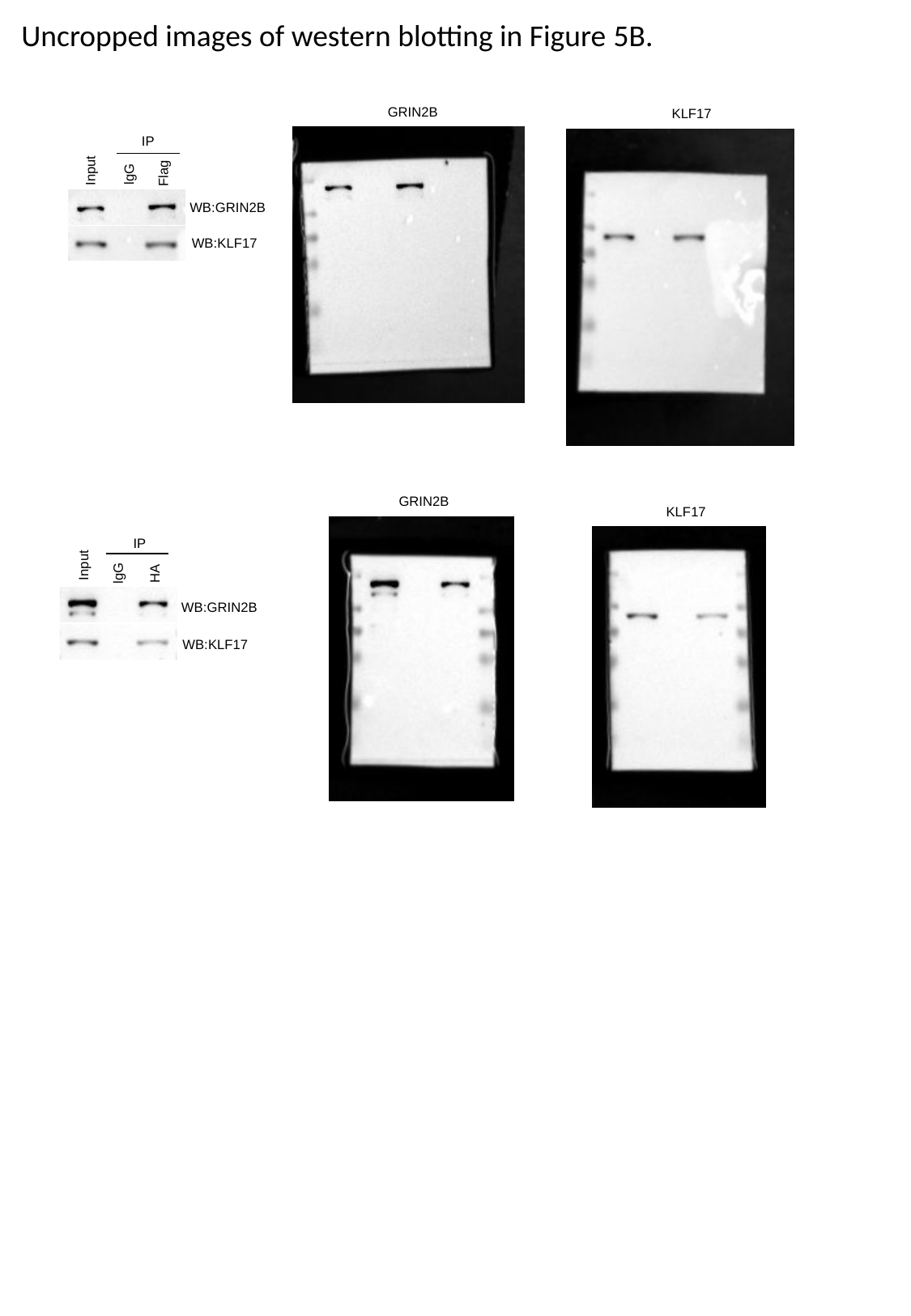

Uncropped images of western blotting in Figure 5B.
GRIN2B
KLF17
IP
Input
Flag
lgG
WB:GRIN2B
WB:KLF17
GRIN2B
KLF17
IP
Input
lgG
HA
WB:GRIN2B
WB:KLF17

## Slide 8
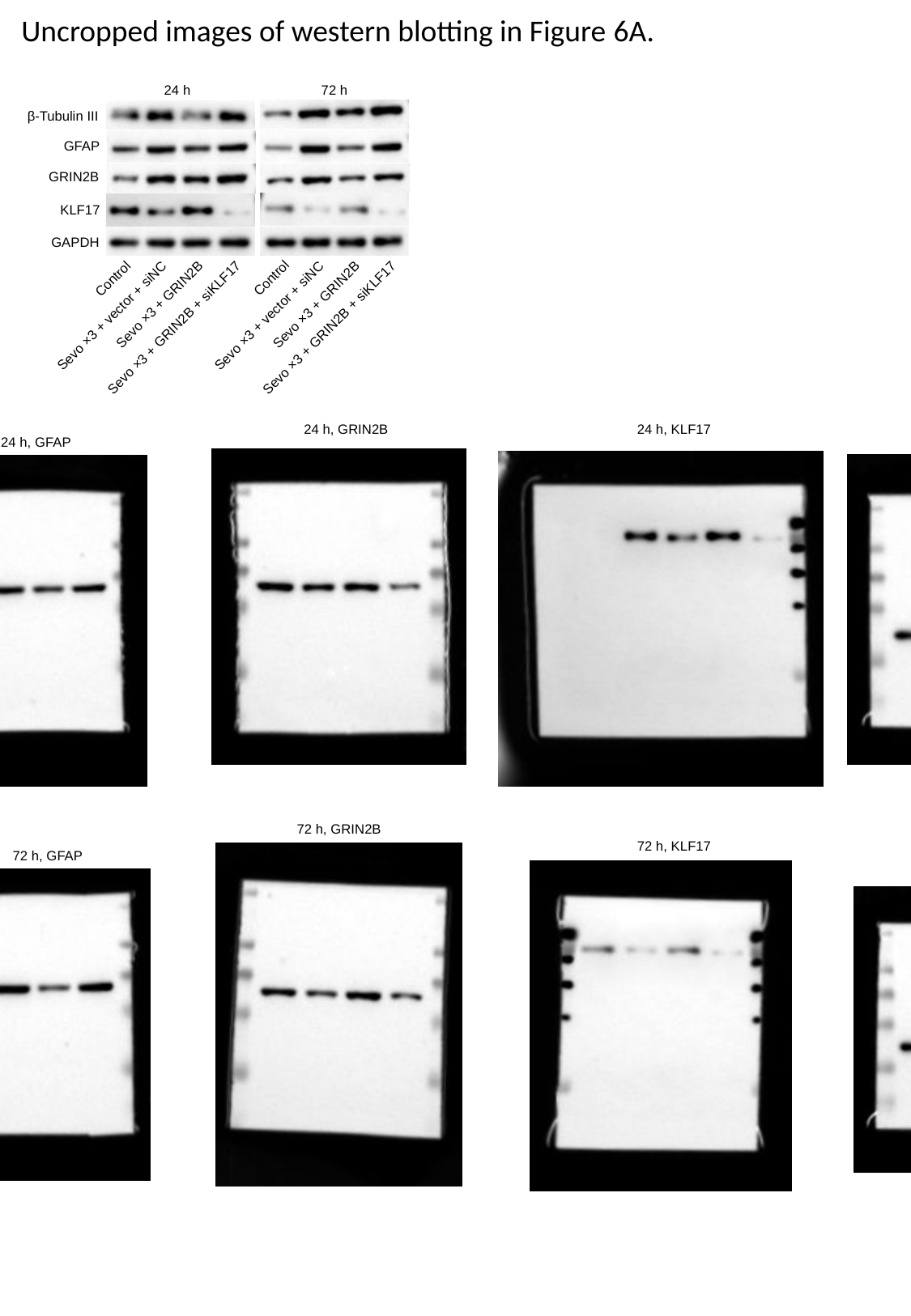

Uncropped images of western blotting in Figure 6A.
72 h
24 h
β-Tubulin III
GFAP
GRIN2B
KLF17
GAPDH
Control
Control
Sevo ×3 + GRIN2B
Sevo ×3 + GRIN2B
Sevo ×3 + vector + siNC
Sevo ×3 + vector + siNC
Sevo ×3 + GRIN2B + siKLF17
Sevo ×3 + GRIN2B + siKLF17
24 h, GRIN2B
24 h, KLF17
24 h, GAPDH
24 h, β-Tubulin III
24 h, GFAP
72 h, GRIN2B
72 h, KLF17
72 h, β-Tubulin III
72 h, GFAP
72 h, GAPDH
